# Automatic Detection of Brain Tumor on Computed Tomography Images for Patients in the Intensive Care Unit

**DOI:** 10.1155/2020/2483285

**Published:** 2020-07-14

**Authors:** Fahmi Fahmi, Fitri Apriyulida, Irina Kemala Nasution, Sawaluddin

**Affiliations:** ^1^Department of Electrical Engineering, Faculty of Engineering, Universitas Sumatera Utara, Medan, Indonesia; ^2^Department of Neurology, Faculty of Medicine, Universitas Sumatera Utara, Medan, Indonesia; ^3^Faculty of Computer Science and Information Technology, Universitas Sumatera Utara, Medan, Indonesia

## Abstract

Patients in the intensive care unit require fast and efficient handling, including in-diagnosis service. The objectives of this study are to produce a computer-aided system so that it can help radiologists to classify the types of brain tumors suffered by patients quickly and accurately; to build applications that can determine the location of brain tumors from CT scan images; and to get the results of the analysis of the system design. The combination of the zoning algorithm with Learning Vector Quantization can increase the speed of computing and can classify normal and abnormal brains with an average accuracy of 85%.

## 1. Introduction

Patients in the intensive care unit require fast and efficient handling, including in-diagnosis service. The development of technological systems in the medical world is now proliferating. Many applications have been built that can process medical image results from modalities such as Computed Tomography (CT), Magnetic Resonance Imaging (MRI), Positron Emission Tomography (PET), and X-ray systems [1]. In the medical world, CT scans are widely used to support the diagnosis of a disease that can display body tissue without the need to go through surgery.

Detection of brain tumors has an essential role in the field of biomedical application in terms of diagnosis of medical image records. The importance of identifying brain tumors has increased in the recent years. The brain tumor classification was developed to help medical staff diagnose the disease. In the classification, there are several processes that need to be performed, e.g., preprocessing, feature extraction, and classification. Preprocessing is part of processing an image before feature extraction is performed to determine an area or object. This process consists of filtering, normalizing, and identifying objects before the extraction stage. Feature extraction is a step to take the core value (feature) on a CT-scan image to get an object that will be recognized or distinguished from other objects [2]. The extraction features include using the Gray Level Co-Occurrence Matrix (GLCM) with a matrix size of 64 × 64 pixels [3], with discrete wavelet transform applying Principal Component Analysis (PCA) [4] or zoning method [5].

Classification is the process of determining functions that can distinguish concepts with the aim of estimating the unknown class of an object [6]. One method that can be used in the classification is Learning Vector Quantization (LVQ). LVQ is a classification method that can conduct training at supervised layers of a version of the Kohonen model that has a simple learning algorithm consisting of one input and output layer [7].

The following are several studies that have been previously conducted in the area of brain tumors, including the classification of soft tissues within brain CT scan based on wavelet-dominant gray level run length texture features [8]. This study obtained standard brain accuracy and brain tumor identification of 98.00%. Classification of brain tumors based on the statistical feature set uses the support vector machine with an accuracy of 68.1% [9].

In this study, we used feature extraction with zoning and classification methods with Learning Vector Quantization (LVQ) techniques. For each sample data that has gone through the image preprocessing process and feature extraction, and tumor location will be determined.

The objectives are to produce a computer-aided system so that it can help radiologists to classify the types of brain tumors suffered by patients quickly and accurately; to build applications that can determine the location of brain tumors from CT scan images, and to get the results of the analysis of the system design. The benefits obtained from this study are to help radiologists to diagnose the types of brain tumors suffered by patients quickly and accurately, especially patients in the intensive care unit, and also, it becomes one of the references for researchers who focus on computer vision technology in the medical field.

## 2. Materials and Methods

### 2.1. Brain Tumors

The brain tumor is tissuemass that grows uncontrollably and is suppressing other healthy tissue. Brain tumors can be classified as benign (soft) brain tumors and malignant (severe) brain tumors. It is clinically challenging to distinguish between benign or malignant brain tumors because the symptoms that arise are also determined by the location of the tumor, the rate of growth, and the effect of the tumor mass on brain tissue [10].

Initial diagnosis is made by obtaining data on the patient's family health history and physical examination. After that, a neurological examination is performed to determine the mental status, memory, cranial nerve function, muscle strength, and response to pain. The next step is a radiological examination through CT-scan or MRI (Magnetic Resonance Imaging).

### 2.2. Computed Tomography

**Figure 1:**
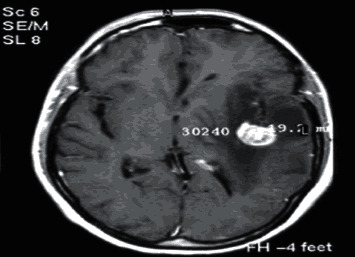
Brain tumor on CT-scan [12].

**Figure 2:**
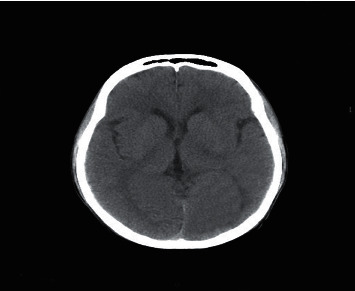
Normal brain on CT-scan.

Computed Tomography (CT) scan is a method used to examine patients without direct surgery but uses X-ray and a computer to produce brain images in axial fragments [11]. The number of pieces produced by the CT-scan is determined by the specifications of the CT-scan used. In order to improve the quality of the image in the radiological examination, the patient is sometimes injected with a contrast agent in order to improve the image quality of the desired organ. The size of the images contained on the CT-scan ranges from −1024 to +3071 on the Hounsfield unit scale. Hounsfield itself is a measurement of the density of the tissue, as shown in Figures 1 and 2.

Image processing is a method for processing and analyzing images so as to produce images in accordance with image perceptions and needs to be used using computer aids. The image can be interpreted as a function that has two dimensions *f* (*x*, *y*), where *x* and *y* are coordinates and *f* at each point (*x*, *y*) expresses the intensity, brightness, and grayscale in the image. The digital image is the study of a matrix in which there are elements of an image that can provide information in a discrete form. Digital images are continuous as in X-ray and television monitors. Thus, the conversion process needs to be performed to get information from the required digital image. To get feature information on images, various applications can be used, one of which is computer vision, which has been developed in the process of taking image information in the form of features that have been extracted automatically from the image itself. This process is often used to combine several technologies such as image and signal processing, pattern recognition and multimedia, and interaction between humans and computers. This process is often referred to as CBIR (Content-Based Image) in the field of image processing [12].

Some stages contained in the CBIR process include the following:

  (1) Preprocessing aims to determine an object that will be used at the extraction stage
  (2) Feature Extraction is a process for obtaining new features in the form of patterns, shapes, and textures

### 2.3. Forming a Binary Matrix (Binarization)

At this stage, the image will be formed as black and white by converting a gray-level image to a binary image. This process will take the average value of each RGB value, where the pixel value produced is higher than the threshold value, it will be represented aswhite and if the resulting pixel value is less than the threshold value, it will be represented as black [13]. The thresholding process is used to determine the degree of the gray level in the image and determine the threshold value. The process for determining this threshold value uses the following equation:

(1) $$\begin{array}{c} T=\frac{f\text{maks}+f\min}{2}, \end{array}$$

where *T* = threshold value, *f*maks = maximum pixel value, and *f*min = minimum pixel value.

### 2.4. Feature Selection

Feature selection is the process of determining patterns by obtaining values on image characters to form feature values. Classification uses feature values to recognize input units from output units so that they can easily distinguish objects.

### 2.5. Zoning Method

**Figure 3:**
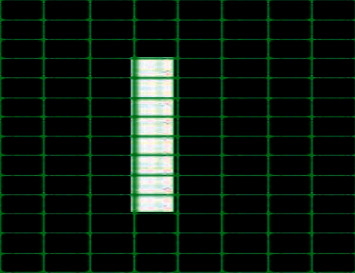
Zoning method.

The zoning method is a method of feature extraction that can divide the characters into *N* × *M* zones from each zone. The feature value calculation is performed to form the feature values in the *M* × *N* zone. In the classification process, the introduction of the zoning method produces proper and efficient feature extraction. Zoning can be used to calculate the number of white pixel values in a particular zone; the value obtained from the zoning process will be used as a value for the vector input. The results of the zoning process are vector features that can be entered into the classification stage, as in Figure 3 [14].

### 2.6. Definition of LVQ (Learning Vector Quantization)

**Figure 4:**
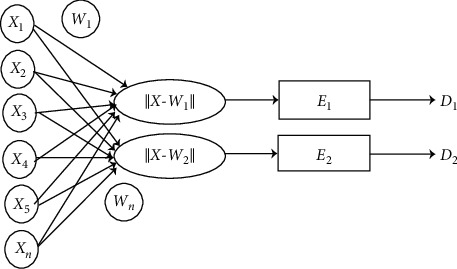
LVQ network architecture [15].

LVQ is a classification method that can conduct training at supervised layers at the competitive layer. This layer is able to classify the given vector input automatically. Some input vectors have close weights; therefore, the weights will connect the input layer with the competitive layer. The competitive layer produces a class that is connected to the output layer with an activation function. The architecture of an LVQ network with several input layer units and units at the output layer can be seen in Figure 4 [16].

Figure 4 explains that the values *X*1 to *X*2 are input values where this value will be used for the training process and testing process. *W*1 and *W*2 as weight vectors that can connect each input layer with the output layer. *W*1 and *Wn* are used to get the smallest weight distance from the weight vector obtained from the calculation of input values. *E*1 and *E*2 are used as the output layer to represent several classes, while *D*1 and *D*2 are used as the output values at the output layer for the testing process.

Some of the advantages of LVQ are as follows [16, 17]:

1. Able to produce a minimum error value
2. At the classification, the stage can summarize large data sets into small vectors
3. Can do a gradual renewal of the resulting model

The disadvantages of LVQ are as follows:

1. To determine the distance to all attributes, an accurate calculation must be used.
2. Calculation of initialization and parameters are needed in determining the accuracy of the LVQ model.
3. There is difficulty in determining the number of vectors in new problems before entering into the classification process using the LVQ method, the training process is first carried to simplify the process of class searching so that it can perform an introduction of input patterns based on the output obtained. LVQ can perform input pattern recognition if the distance between the weight vector and the input vector is close together.

### 2.7. Training and Testing

At LVQ, there are two stages of training and testing as follows: the training algorithm and testing of the LQV artificial neural network used for the training and testing process . The initial weight of the input values *X*1 to *Xn* towards the output layer that represents the whole class, maximum epoch (MaxEpoch), learning rate parameters (*α*), reduction of learning rate (Dec*α*), and minimum error (Eps) is determined.

At the training stage, LVQ calculation results are used to get the weight value that will be stored and used in the testing phase. In the testing phase, new input data is classified by calculating the value of each weight in the input and selecting the smallest distance in the two weights that have been stored. The value at the smallest weight distance will represent the class in the input image.

The data used are 40 data stored in the medical record of H. Adam Malik General Hospital in patients aged 40 to 60 years with male sex 60% and female 40%, all anonymized.

### 2.8. Input of the Brain Image

The image input process is carried out before the image classification process. The image data used in this study is axial piece brain image data obtained from CT Scan. The image used is a grayscale image measuring 512 × 512.

### 2.9. Preprocessing

**Figure 5:**
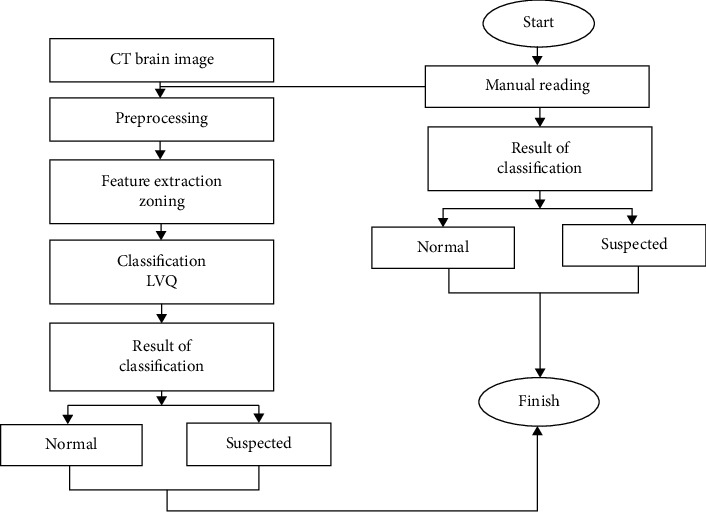
Research flowchart.

**Figure 6:**
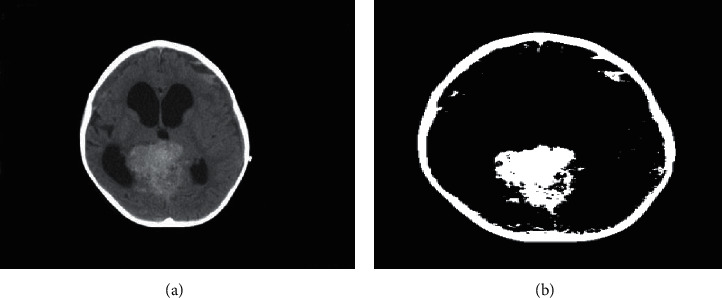
Binarization, normal (a) and after binarization (b).

At the preprocessing stage shown in Figure 5, several stages are carried out to facilitate the next process; the preprocessing stage consists of grayscale and binarization processes. At the stage of binarization, the image is converted into a grayscale form, and then, thresholding will be performed where the grayscale image will be converted into a binary form, which has values 1 and 0 (white and black). In this stage, the threshold value is used to determine the binary value in each image. If the resulting value is above the threshold value, the pixel value is changed to white; if the resulting value is less than the threshold, the pixel value will be changed to black. This is shown in Figure 6.

### 2.10. Feature Extraction

In the next process, after going through the preprocessing, the feature extraction step is carried out by the zoning method to get a good feature value on the image of a brain tumor. Furthermore, the feature values obtained from the method will be classified using the LVQ (learning vector quantization) method.

**Figure 7:**
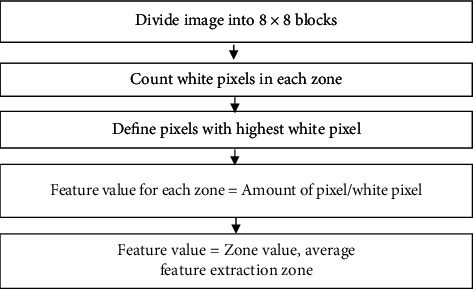
Feature extraction process.

**Figure 8:**
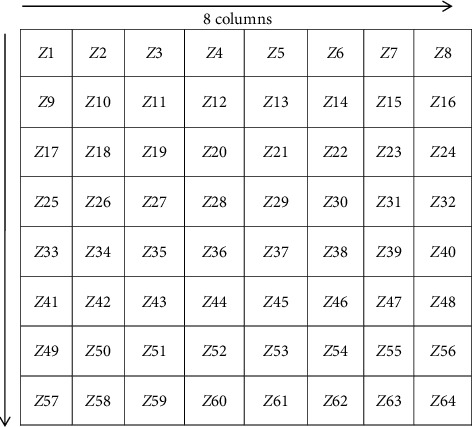
Result of image zoning.

Zoning is one method that can divide several regions, where each region will produce a feature value by counting the highest number of white pixels. At this stage, the image size of 512 × 512 will be divided into 8 columns and 8 rows so that it gets 64 zones, and there are 64 feature values in it. The following process of extraction features can be seen in Figure 7, while the division of zones can be seen in Figure 8.

The process of the zoning method on CT brain image tumors is as follows:

1. The number of white pixels are counted per zone from *Z*1 to *Z*512
2. It is determined which zone has the highest number of white pixels
3. The feature values (*Zn*) of each zone are calculated from *Z*1 to *Z*512

The following formula is used:

(2) $$\begin{array}{c} Zn=\frac{Zn}{Z\text{ highest}}, \end{array}$$

where 1 ≤ *n* ≤ 512.

Feature values (*Zn*) are obtained by comparing the number of white pixels from one zone with the zones obtained from process no 2. Examples of zoning method calculations are as follows:

(1) The number of white pixels in each zone is
    Z4 = 40, *Z*12 = 30, *Z*40 = 70, and *Z*53 = 50
(2) The zone that has the highest number of white pixels is *Z*40 = 70
(3) Feature values for each zone include
   
*Z*1 = 40/70 = 0.57
   
*Z*12 = 30/70 = 0.42
   
*Z*40 = 70/70 = 1
   
*Z*53 = 50/70 = 0.71
   
*Z*60 = 20/70 = 0.28

**Figure 9:**
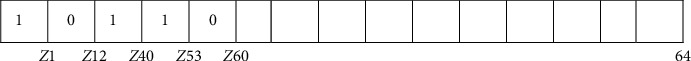
Value of feature extraction with zoning.

The feature extraction process by the zoning method will produce 64 features where the feature will be used as an input value at the next stage, which is the classification process using LVQ and can be seen in Figure 9.

### 2.11. Classification

Classification is a process to train and test the value of features produced through the feature extraction process using the zoning method and classified with Learning Vector Quantization (LVQ). At the classification stage, there are two processes, namely, the training process and the testing process, where the training process is used to train memorization on Learning Vector Quantization (LVQ) while the testing process is testing the value of features that have never been trained [15].

### 2.12. Training Process

At the training stage, the LVQ algorithm will process the input values by receiving 64 input vectors in the feature class, and then, the vector will calculate the distance of all vectors representing the class.

The process of applying the Vector Quantization Learning Algorithm (LVQ) to the training is as follows [18]:

1. The initial process in the LVQ algorithm is the initialization stage to determine the initial weight, maximum iteration, minimum error, and learning rate.
2. The input and target values of the input are initialized.
3. The next step determines the initial conditions epoch = 0 and error = 1.
4. When the epoch is smaller than the maximum epoch, each weight value is calculated, and then, the shortest distance to the weight is set with the value that has been set.
5. The next step is to update the weight value if the target class and weight are the same as when using the following equation:

(3) $$\begin{array}{c} wj\left(\text{new}\right)=wj\left(\text{old}\right)+\alpha \left[x-wj\left(\text{old}\right)\right]. \end{array}$$

(6) The same calculation is repeated for each input using the updated weight.
(7) After the calculation of the input is complete, the value of *α* is reduced and *α* is iterated until it approaches the maximum. The value is determined so that Error ‖*X* − *Wj*‖ becomes a minimum.

### 2.13. System Testing and ROC Analysis

The testing process uses LVQ that has been trained to recognize test data that has never been trained. The testing process is the same as the training process, where the classification calculates the value of each weight input and selects the closest distance between the two weights. When LVQ is tested using training data, testing is performed to see how the memorization of LVQ is developed after going through the training process because the cases included have been studied before.

The results of the testing process on the sample will be adjusted in the contingency table to get the sensitivity, specificity, and accuracy of the contingency table, as can be seen in Table 1. The probability of success from a calculation has four possibilities. The four possibilities are as follows:

1. If the doctor diagnoses the brain image as a tumor and the brain image is classified positively identified by the tumor, then true positive (TP) is calculated
2. If the doctor diagnoses the brain image as a tumor and the brain image is classified as negatively identified by the tumor, then false negative (FN) is calculated
3. If the doctor diagnoses a healthy brain and the brain image is classified as negative, true negative (TN) is calculated
4. If the doctor diagnoses a healthy brain image and the brain image is classified as positive, the tumor is calculated as false positive (FP)

(4) $$\begin{array}{c} \text{TPR}=\frac{\text{TP}}{\text{P}}=\text{Recall,} \end{array}$$

(5) $$\begin{array}{c} \text{FPR}=\frac{\text{FP}}{\text{N}}, \end{array}$$

(6) $$\begin{array}{c} \text{Precision}=\frac{\text{TP}}{\text{TP}+\text{FP}}\;\times 100\%, \end{array}$$

(7) $$\begin{array}{c} \text{Accuracy}=\frac{\text{TP}+\text{TN}}{\text{P}+\text{N}}\;\times 100\%, \end{array}$$

(8) $$\begin{array}{c} \text{Sensitivity}=\frac{\text{TP}}{\text{TP}+\text{TN}}\;\times 100\%. \end{array}$$

### 2.14. Image Preparation

This part discusses the process of classifying brain images, amounting to 40 images. In the training process, 10 brain images were identified as tumors, and 10 healthy brains for the training process consisted of 10 brain images that were identified as tumors, and 10 were normal. CT-scan brain image data were obtained from medical records of H. Adam Malik General Hospital in patients aged 40 years to 60 years. At this stage, it aims to display the results of the testing process on learning vector quantization. The author builds this application using programming in java.

The CT image data used to support the findings of this study may be released upon application to the Department of Neurology, Adam Malik Hospital, Medan, Sumatera, Utara, Indonesia, who can be contacted at irina.kemala (at) usu.ac.id.

### 2.15. Specifications of the CT-Scan Plane Used

The CT-scan aircraft specifications used are as follows:

1. Brand: GE LightSpeed 16 Slice CT
2. Rotation: 0.5 s
3. Thickness slice: 5 mm
4. Kv: 120 kV

## 3. Results and Discussion

### 3.1. Preprocessing

**Figure 10:**
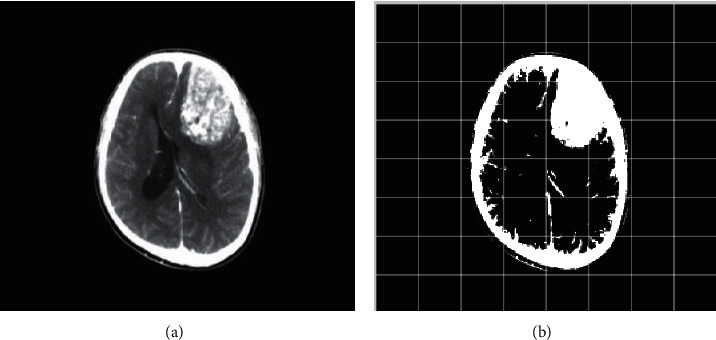
Binary image reconstruction.

The initial process carried out in the classification of brain tumors through the stages of preprocessing in which the image will be converted into black and white before the globalization process is carried out. In this study, using a threshold value of 128, determining the threshold value is used to obtain gray values in the image of a brain tumor. The results of this process can be seen in Figure 10.

After the binary image is obtained, the next step is feature extraction using the zoning method. In addition to determining the value of the zoning method, features are also used to determine the location of the tumor. A division of several zones of the same size is performed in order to get the results of the CT scan brain tumor image values. The purpose of this feature extraction stage is to find a collection of features found in the character of the brain image.

### 3.2. Training Process

**Figure 11:**
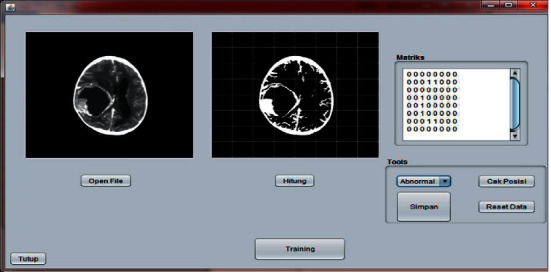
Training process.

The image training process uses data in the form of 20 CT scan brain images consisting of 10 normal images and 10 suspected images. CT scan brain images used are 512 × 512 in size stored on local hard disks, and the classification process uses an artificial neural network LVQ (Learning Vector Quantization). Training can be seen in Figure 11, while the training data from CT brain image can be seen in Table 2.

From Table 2, the weight value in each training data has been seen. The weight value is the final value used in the testing process, where the weight value is obtained from the LVQ calculation process. To make an introduction to the image by calculating the value of each weight in the input and choose the smallest distance on both weights. The value at the smallest weight distance will represent the class in the input image.

### 3.3. Image Testing Process

**Figure 12:**
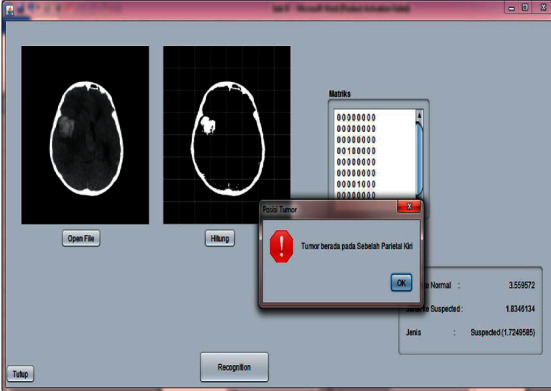
Image testing process.

During the testing phase, 20 imagery input data were used, consisting of 10 normal brain images and 10 suspected brain images. Display application of the classification of brain tumors by zoning using learning vector quantization can be seen in Figure 12, and the results of the test can be seen in Table 3.

From Table 3, the input value in data-12 shows the condition of the image for the normal category, but the system shows a suspect; this is because the learning vector quantization method has a weakness of being sensitive to changes in weight values. If the position of the input value in the form of a feature value is changed, the weight value will also change.

### 3.4. Classification Results Using the LVQ Method

The results of the test data on the application of brain tumor classification are obtained with the learning vector quantization method with an average classification result of 85% so that the results of the accuracy using learning vector quantization can be calculated simply by using the following equation:

(9) $$\begin{array}{c} \text{accuracy}=\frac{\text{amount of succesful classification}}{\text{amount of total samples}}\times 100\%,\\ =\frac{17}{20}\times 100\%=85\%. \end{array}$$

To get accurate results, it is necessary to perform an ROC analysis of the classification results based on the LVQ method; the results for the LVQ method are shown in Table 4.

This study has several limitations. The use of CT imaging in the intensive care unit is very limited nowadays due to the need for a radiographer's presence to analyze and diagnose the patients, sometimes in a not very regular period of time. The availability of CT data, therefore, was very limited, especially with manual reading as the golden standard, as explained in the research flowchart (Figure 5). In this study, only 20 images from 40 data images are used for training, and the rest 20 images are used for testing purposes. This split portion of training and testing was applied in order to get the proof on a concept and can be extended in the future.

## 4. Conclusions

The combination of the zoning algorithm with learning vector quantization can increase the speed of computing and can classify normal and abnormal brains with an average accuracy of 85%. Optimal recognition of image data can be achieved with the learning vector as it is suitable for use in the intensive care unit in hospitals.

The quantization method has a fast calculation in the introduction of an appropriate character so that there are no errors when testing data. The suggestions for developing this study are to compare the learning vector quantization method with the support vector machine method so that it can produce the best way for classifying brain images. The use of the zoning method and learning vector quantization can be applied to further research by adding methods to determine the extent of abnormal brain images.

## Figures and Tables

**Table 1:** Contingency table.

	P	N
Y	TP (True Positive)	FP (False Positive)
N	FN (False Negative)	TN (True Negative)
Total	P	N

**Table 2:** Data set training.

No.	Brain image	Weight	Result
1	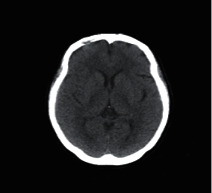	0.076161	Normal
2	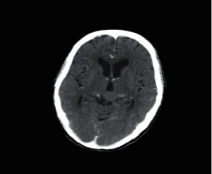	2.304818	Normal
3	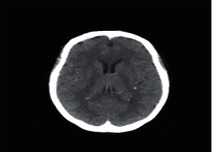	2.2668598	Normal
4	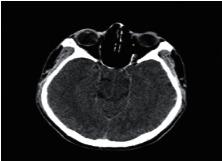	1.456988	Normal
5	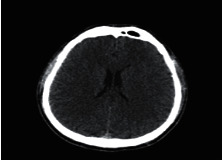	2.9700592	Normal
6	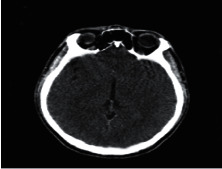	1.6688762	Normal
7	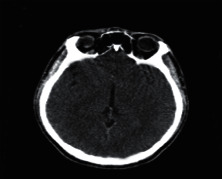	1.6688761	Normal
8	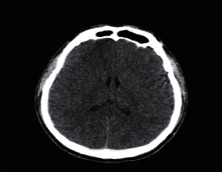	2.9700592	Normal
9	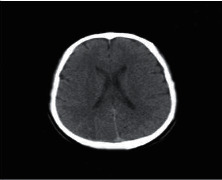	2.769984	Normal
10	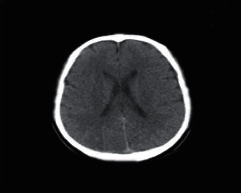	1.6688762	Normal
11	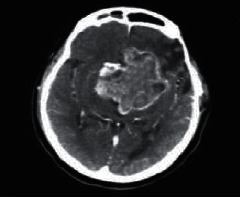	2.9779766	*Suspected*
12	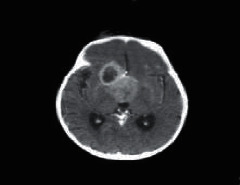	2.9736168	*Suspected*
13	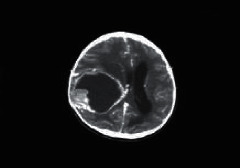	2.9230187	*Suspected*
14	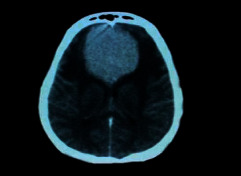	2.800454	*Suspected*
15	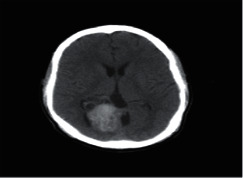	3.2186778	*Suspected*
16	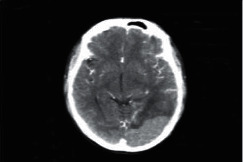	1.8768171	*Suspected*
17	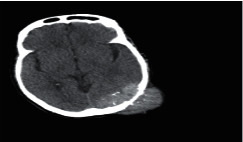	2.3506687	*Suspected*
18	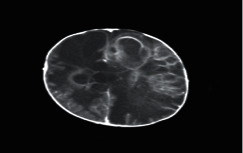	1.9752706	*Suspected*
19	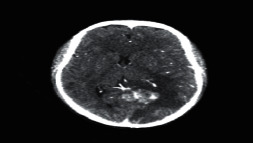	3.0961044	*Suspected*
20	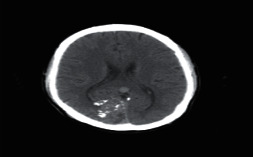	2.006145	*Suspected*

**Table 3:** Result of testings.

No.	Image	Weight 1	Weight 2	Input^∗^	Result	Notes	Pos.
1	Data-5.png	3.489579	2.9736168	*Suspected*	*Suspected*	TP	Right
2	Data-9.png	3.7573445	3.2186778	*Suspected*	*Suspected*	TP	Right
3	Data-2.png	3.4895792	2.9779766	*Suspected*	*Suspected*	TP	Right
4	Data-12.png	3.0037804	2.6869104	Normal	*Suspected*	FN	Normal
5	Data-13.png	2.304818	2.9392818	Normal	Normal	TN	Normal
7	Data-7.png	3.07729601	1.8768171	*Suspected*	*Suspected*	TP	Right
9	Data-19.png	2.2668598	2.9678066	Normal	Normal	TN	Normal
10	Data-11.png	2.305248	2.9393818	Normal	Normal	TN	Normal
11	Data-13.png	2.304818	2.93938118	Normal	Normal	TN	Normal
12	Data-8.png	3.4555268	2.9230187	*Suspected*	*Suspected*	TP	Left
13	Data-18.png	2.9360542	2.8076556	Normal	*Suspected*	FN	Normal
14	Data-4.png	3.5838966	2.800454	*Suspected*	*Suspected*	TP	Right
15	Data-1.png	3.043977	2.006145	*Suspected*	*Suspected*	TP	Right
16	Data-6.png	3.0350804	2.3506687	*Suspected*	*Suspected*	TP	Left
17	Data-10.png	2.3829138	3.0961044	*Suspected*	Normal	FP	Normal
18	Data-3.png	2.8869388	1.9752706	*Suspected*	*Suspected*	TP	Right
19	Data-14.png	2.769984	2.9166098	Normal	Normal	TN	Normal
20	Data-15.png	2.2493293	2.9494078	Normal	Normal	TN	Normal

**Table 4:** LVQ result.

Classification	LVQ
TP	9
TN	8
FP	2
FN	1
Sensitivity %	90
Specificity %	80
Accuracy %	85

## Data Availability

All the brain CT Image data used to support the findings of this study are available from the corresponding author upon request.
